# Mitochondrial Quality Control in Age-Related Diseases: From Molecular Architecture to Precision Therapeutics

**DOI:** 10.3390/antiox15070830

**Published:** 2026-06-30

**Authors:** Jingmin Che, Ye Sun, Fang Wang, Qing Feng, Cuixiang Xu, Xuhui Li

**Affiliations:** 1 Shaanxi Provincial Key Laboratory of Infection and Immune Diseases, Shaanxi Provincial People’s Hospital, Xi’an 710068, China; jm.che1123@mail.nwpu.edu.cn (J.C.); fengqing@spph-sx.ac.cn (Q.F.); 2 Shaanxi Engineering Research Center of Cell Immunology, Shaanxi Provincial People’s Hospital, Xi’an 710068, China; 3 Frontier Institute of Science and Technology, Xi’an Jiaotong University, Xi’an 710049, China; sunye@stu.xjtu.edu.cn; 4 Department of Oncology, Shaanxi Provincial People’s Hospital, Xi’an 710068, China; wangfang1@mail.nwpu.edu.cn; 5 College of Forensic Medicine, Xi’an Jiaotong University Health Science Center, Xi’an 710061, China

**Keywords:** mitochondrial quality control, cellular senescence, mitophagy, age-related diseases, therapeutic targets, metabolic homeostasis

## Abstract

Background: Mitochondria are the primary organelles that regulate cellular bioenergetic metabolism and maintain homeostasis, providing essential structural support for optimal cell survival. Nonetheless, advancing age leads to cumulative damage to mitochondrial structure and functional integrity, which is a defining characteristic of biological aging and is closely linked to the emergence and progression of numerous age-related diseases, including neurodegenerative disorders, cardiovascular diseases, and metabolic disorders. Scope of review: This article offers a thorough summary and review of mitochondrial quality control (MQC), emphasizing numerous critical processes, including mitochondrial biosynthesis, dynamic remodeling (fusion and fission), and mitophagy. We thoroughly elucidate the molecular pathways that regulate MQC and demonstrate how age-related dysregulation precipitates cellular senescence, highlighting the transition from physiological maintenance to pathological malfunction, which ultimately culminates in cellular aging. Conclusions and implications: This study systematically elaborates the pathophysiological mechanisms in the field, comprehensively evaluates the clinical translational potential of targeting the MQC pathway, highlights the key objectives of “restoring mitochondrial plasticity and removing dysfunctional mitochondria”, and explores novel intervention strategies. The restoration of normal mitochondrial function in cells throughout aging is a very promising path for precision medicine therapeutics with great translational potential, according to recent state-of-the-art research. The development of novel therapeutic approaches to improve functional healthy mitochondria can effectively delay aging and reduce the rising global burden of age-related diseases.

## 1. Introduction

The aging global population presents substantial challenges to existing healthcare systems, particularly highlighted by the rising prevalence of chronic conditions such as neurodegenerative disorders, cardiovascular failure, and sarcopenia. Increasing data indicate that mitochondrial dysfunction is not simply a passive result of aging but a fundamental factor that actively accelerates organismal aging [1,2]. Mitochondria, as the metabolic center of the cell, are crucial for energy conversion, redox signaling, and directly affecting cell fate. Mitochondrial homeostasis is essential for maintaining cellular health, a process carefully governed by mitochondrial quality control (MQC) processes [3].

In physiological conditions, MQC functions as an integrated, dynamic monitoring system. It regulates mitochondrial biogenesis and morphological remodeling (fusion/fission dynamics) through the peroxisome proliferator-activated receptor γ coactivator 1-alpha-sirtuin 1 (PGC1α-SIRT1) axis, while concurrently recognizing and removing damaged organelles via targeted mitophagy [4]. Nonetheless, the aging process is often associated with a comprehensive breakdown of this surveillance system. The efficacy of MQC declines with age, as evidenced by diminished bioenergetic signaling and altered mitophagic flux. This deterioration leads to the intracellular buildup of fragmented mitochondria, thus ensnaring cells in a self-sustaining cycle of oxidative stress and cellular senescence.

Contemporary research endeavors have primarily focused on molecular signaling pathways and therapeutic potentials in isolation or have been restricted to individual disease models. This work aims to elucidate the role of dysregulated MQC in the progression of several age-related diseases. Additionally, the potential impact of contemporary therapies, including mitochondrial transplantation and nicotinamide adenine dinucleotide (NAD+) supplementation, on the delay of the aging process is examined in this study. The objective is to implement comprehensive strategies that will alleviate the progressively severe adverse effects of age-related diseases.

## 2. Methods

To thoroughly clarify the mechanisms of MQC in aging across diverse diseases and investigate potential strategies for postponing aging via the targeted modulation of mitochondrial quality control, we performed a systematic literature review encompassing primary biomedical databases such as PubMed/Medline, Web of Science, Scopus, and the Cochrane Library. The research utilized a retrieval strategy that merged Boolean operators (AND, OR, NOT) with Medical Subject Headings (MeSH), encompassing three principal concepts: (1) fundamental MQC mechanisms (including mitochondrial synthesis, the mitotic chaperone system, dynamic mitochondrial regulation, mitochondrial autophagy, and mitochondrial translocation); (2) age-associated diseases (including neurodegenerative disorders, cardiovascular conditions, metabolic diseases, and cancers); and (3) translational medicine approaches (such as small-molecule inhibitors and biomarkers). Secondly, we conducted a manual assessment of the reference lists from the selected papers to confirm that no essential research was overlooked. The inclusion criteria for literature were tightly confined to original research articles, systematic reviews, and meta-analyses published in English and subjected to peer review.

## 3. Integrated Molecular Framework of the Mitochondrial Quality Control Network

**Figure 1:**
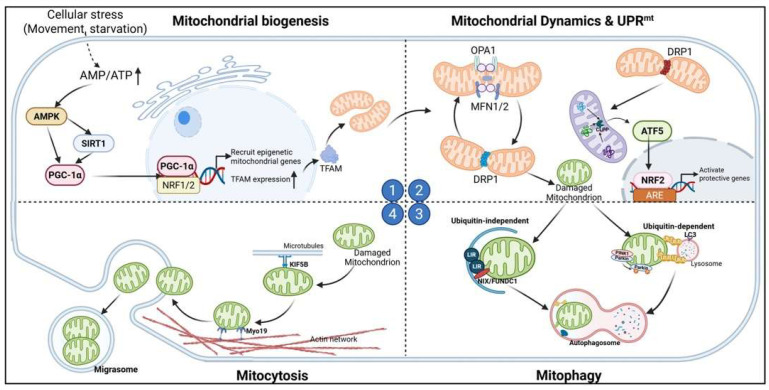
The fundamental mechanism of mitochondrial quality control (MQC) for preserving cellular homeostasis. This model outlines four coordinated methods of mitochondrial quality regulation. Cellular stress, such as physical exertion or starvation, increases the AMP/ATP ratio and activates the AMPK/SIRT1–PGC-1α signaling pathway, which works in conjunction with NRF1/2 to promote TFAM expression and augment mitochondrial biogenesis. Mitochondrial fusion and fission are regulated by OPA1, MFN1/2, and DRP1, enabling network reorganization and the separation of dysfunctional mitochondria. Mitochondrial stress activates UPR^mt^ through ATF5 and NRF2/ARE signaling, resulting in the expression of protective genes. Defective mitochondria are removed through mitophagy via NIX/FUNDC1–LC3-mediated ubiquitin-independent pathways or PINK1/Parkin-mediated ubiquitin-dependent pathways. Moreover, dysfunctional mitochondria can be transported along microtubules and actin filaments by KIF5B and Myo19, and later eliminated via mitocytosis into migrasomes. These systems collectively maintain mitochondrial homeostasis and cellular life. Created in BioRender (2026). 18 June 2026. https://BioRender.com/dlmsfxh.

The regulation and maintenance of mitochondrial homeostasis are governed by a complex, hierarchical interconnected network. This network regulates critical activities such as mitochondrial biosynthesis, dynamic remodeling, and autophagy, and adeptly translates metabolic information into mitochondrial adaptive responses via unique post-translational modifications and interorgan communication [5,6] (Figure 1).

### 3.1. Mitochondrial Biogenesis

Mitochondrial biogenesis is a dynamic process involving the utilization of pre-existing mitochondria by cells to generate new ones. This mechanism requires synchronized regulation between the nuclear genome and the mitochondrial genome to maintain homeostasis in mitochondrial quantity, function, and cellular energy [7,8].

The regulation of mitochondrial biogenesis constitutes a multi-level cascade network, with its core primarily mediated by the transcription co-activator PGC-1α [9]. The activated PGC-1α enhances its transcriptional activity by binding to nuclear respiratory factors (NRF-1 and NRF-2) and further recruiting epigenetic enzymes, thereby inducing the transactivation of various nuclear-encoded mitochondrial structural and functional genes by the NRFs. Most critically, it upregulates the expression of mitochondrial transcription factor A (TFAM), which promotes mtDNA replication and the transcription of core subunits involved in oxidative phosphorylation, thereby facilitating mitochondrial biogenesis [8,10,11]. This metabolic process is closely linked to the cellular bioenergetic signaling pathway involving adenosine monophosphate (AMP)-dependent protein kinase (AMPK) and SIRT1. When cells enter an energy-deficient state due to movement, starvation, or metabolic stress, the elevated AMP/ATP ratio promotes the activation of AMPK, which directly facilitates the phosphorylation of PGC-1α, thereby enhancing its transcriptional activity, and AMPK increases mitochondrial biosynthesis by reducing the repressive influence of transcription factor EB (TFEB) via mechanistic target of rapamycin complex 1 (mTORC1) contact inhibition [12,13,14,15]. Recent studies demonstrate that AMPK directly phosphorylates five conserved serine residues in folliculin interacting protein 1 (FNIP1), thereby inhibiting the RagC GAP activity of the folliculin (FLCN)–FNIP1 complex. This alteration facilitates the detachment of mTORC1 from the lysosomal membrane, resulting in the nuclear translocation of TFEB, even in the presence of sufficient amino acids. Consequently, this signaling cascade enhances the expression of genes related to mitochondrial biogenesis [16]. Simultaneously, AMPK also enhances intracellular NAD+ levels by phosphorylating the NAMPT enzyme, thereby indirectly activating the deacetylase SIRT1, which enhances PGC-1α activity via deacetylation. This dual regulatory system satisfies the energy requirements of cellular metabolism by enhancing mitochondrial biosynthesis [17]. Furthermore, calcium ions (Ca^2+^) function as an essential reverse signaling molecule. A reduction in mitochondrial membrane potential (Δψm) diminishes the mitochondria’s capacity to sequester Ca^2+^, resulting in an elevation of cytoplasmic Ca^2+^ concentration. The elevated cytoplasmic Ca^2+^ concentration modulates the expression and transcriptional activity of PGC-1α via activating Ca^2+^-dependent kinases, such as CaMKII and PKC, as well as the phosphatase calmodulin kinase [18].

### 3.2. Mitochondrial Unfolded Protein Response, UPR^mt^

The mitochondrial unfolded protein response (UPR^mt^) is a retrograde signaling system activated by disturbances in mitochondrial proteostasis. When physiological or pathological stressors disrupt the balance between the synthesis of proteins encoded by mitochondrial DNA (mtDNA) and those encoded by nuclear DNA (nDNA), it leads to the abnormal accumulation of unassembled protein subunits within the mitochondrial matrix. Additionally, with aging, the excessive generation of ROS in mitochondria can cause oxidative damage to mitochondrial proteins. This damage can alter their original spatial conformation, induce protein denaturation, and ultimately contribute to the formation of various protein aggregates. Therefore, excessive accumulation of misfolded or not assembled proteins in the mitochondrial matrix caseinolytic protease P (CLPP) degrades them into peptide fragments, which are then transported to the intermembrane space by the inner membrane ABC transporter HAF-1 [19]. Consequently, these signals facilitate the nuclear translocation of the transcription factor activated transcription factor atfs-1 (ATFS-1) (or its human equivalent, ATF5), resulting in the transcriptional upregulation of nuclear genes that encode mitochondrial molecular chaperones, including HSP60 and HSP10, along with proteases [20]. Mitochondrial reactive oxygen species (mtROS) function as signaling molecules that trigger an antioxidant response mediated by NFE2L2 (NRF2). Activated NFE2L2 increases its expression and stimulates mitochondrial biosynthesis via attaching to the antioxidant response element (ARE) in the NRF1 promoter [21,22]. Furthermore, ATF4, CHOP, and C/EBPβ also participate in the regulation of this pathway; phosphorylation of eIF2α promotes the translation of CHOP, ATF4, and ATF5 mRNA, thereby amplifying the UPR^mt^ signaling pathway [21,22].

### 3.3. Mitochondrial Dynamics

Mitochondria function as a highly dynamic regulatory network within cells. The equilibrium between the processes of mitochondrial fusion and fission determines the stability of this dynamic network. Recent study has concentrated on the mechanisms of mitochondrial fusion and fission as well as the regulatory networks controlling connections between various organelles.

Mitofusin 1/2 (MFN1/2) on the outer mitochondrial membrane mediates mitochondrial fusion; optic atrophy 1 (OPA1) on the inner membrane mediates inner membrane fusion [23,24]. Through their GTPase domains, MFN1 and MFN2 facilitate homotypic or heterotypic dimerization, which connects neighboring mitochondria and encourages the fusing of the outer mitochondrial membrane [25]. Thereafter, the proteolytic cleavage of OPA1 splice variants by the proteases OMA1 and YME1L produces the active forms required for the fusing of the inner mitochondrial membrane [26,27]. Mitochondrial fission is predominantly regulated by the cytosolic dynamin-related protein DRP1. DRP1 is attracted to the mitochondrial surface by receptor proteins such as MFF, MID49/51, and FIS1. Upon recruitment, it forms helical structures around the constriction sites and employs GTP hydrolysis to facilitate membrane scission, ultimately resulting in mitochondrial division [28,29]. The processes of mitochondrial fusion and division do not occur in isolation but are precisely regulated to maintain dynamic homeostasis.

The maintenance of mitochondrial dynamic equilibrium relies on the coordinated regulation of multiple molecular mechanisms. PINK1/Parkin enhances the proteasomal destruction of MFN2 during mitochondrial fusion by promoting its ubiquitination, therefore aiding in the removal of damaged mitochondria. The deubiquitinating enzyme USP30 counteracts Parkin’s actions by removing ubiquitin chains from MFN1 and MFN2, therefore preserving mitochondrial integrity [30,31]. DRP1, the principal regulator of mitochondrial fission, relies on a multi-site phosphorylation process for its action. Phosphorylation of DRP1 at Ser637 by PKA obstructs its translocation to the mitochondria, thereby impeding the fission process. Conversely, calcineurin-mediated dephosphorylation at this locus facilitates mitochondrial fission. Furthermore, during mitosis, CDK1 phosphorylates DRP1 at Ser616, promoting mitochondrial fragmentation [32,33,34]. Moreover, SUMOylation is essential for the regulation of DRP1 stability. Research has shown that SENP3-facilitated deSUMOylation improves the mitochondrial translocation efficiency of DRP1 [35]. The endoplasmic reticulum-mitochondrial contact sites (MAMs) serve as critical structural hubs for interorganelle communication. Within these microdomains, the endoplasmic reticulum pre-suction sites regulate actin polymerization through INF2, thereby facilitating the mitochondrial translocation of DRP1 [36]. Furthermore, Bcl-2 family proteins directly regulate mitochondrial fusion and fission dynamics. Bcl-xL selectively binds to the C-terminal domain of DRP1 via its BH4 domain, thereby diminishing DRP1′s GTPase activity and its translocation to the mitochondria. This activity opposes excessive mitochondrial fragmentation and maintains network integrity [37,38,39]. Conversely, the pro-apoptotic protein Bax has been shown to facilitate fission site selection by building complexes with DRP1 and MFN2, a process that is independent of Bax-induced mitochondrial pore development [40,41]. Additionally, Bcl-xL directly interacts with MFN1 and MFN2, augmenting their GTPase activity to facilitate outer mitochondrial membrane fusion [42]. Conversely, Mcl-1 associates with OPA1 to preserve inner mitochondrial membrane fusion and cristae structure; thus, Mcl-1 knockdown cells demonstrate abnormal OPA1 processing and cristae enlargement [43].

### 3.4. Mitophagy

When mitochondrial damage exceeds the reparative and fusion systems’ compensatory capacity, cells initiate mitophagy to remove severely damaged mitochondria. Mitophagy is driven synergistically by two complementary pathways—ubiquitin-dependent and ubiquitin-independent—to ensure the selective removal of damaged mitochondria.

Mitophagy mostly relies on the PINK1-Parkin pathway, which is dependent on ubiquitin. In functional mitochondria, PINK1 (PTEN-induced kinase 1) is translocated to the inner mitochondrial membrane through the TOM/TIM complex, where it is swiftly degraded after cleavage by the PARL protease [44]. Upon the loss of Δψm, PINK1 stabilizes on the outer mitochondrial membrane, accumulating at the TOM complex, where it undergoes autophosphorylation and activation [45]. Activated PINK1 phosphorylates ubiquitin (at Ser65) and the ubiquitin-like domain of Parkin (at Ser65), thereby synergistically recruiting and activating the cytosolic E3 ubiquitin ligase Parkin [46]. The activated Parkin protein promotes the assembly of polyubiquitin chains at lysine 63 (Lys63) and Lys48 residues on mitochondrial outer membrane proteins, including MFN1/2, VDAC, and TOM20 [47]. These ubiquitin chains are identified by autophagy receptors such as p62/SQSTM1, OPTN, NDP52, and TAX1BP1, which then associate with LC3 on the autophagosome membrane. This connection enables the localization of damaged mitochondria to autophagosomes, thus finalizing the mitophagy process [46,48]. Recent research reveals that OPTN and NDP52 function as principal downstream autophagy receptors for Parkin, with their activity further enhanced by TBK1-mediated phosphorylation, establishing a positive feedback loop that improves mitophagy efficiency [49,50]. The ubiquitin-independent process is directly facilitated by intrinsic mitochondrial membrane proteins. Proteins including NIX/BNIP3L, FUNDC1, and BCL2L13 engage with LC3 via their LIR motifs to commence the autophagic process, a mechanism additionally enhanced by HIF-1α under hypoxic conditions [51,52]. Furthermore, other proteins located in the inner membrane and cristae, including FKBP8, Prohibitin 2, and PHB2, have been identified as receptors that promote mitophagy [53].

AMPK has a crucial coordinating role in mitophagy. AMPK promotes the recruitment of DRP1 to the mitochondrial surface by directly phosphorylating mitochondrial fission factor (MFF) at Ser155 and Ser172, leading to mitochondrial fragmentation. This fragmentation generates substrates of appropriate dimensions for mitophagy and enables phosphorylated MFF to serve as a signaling platform for the recruitment of autophagy receptors [54,55]. Cellular energy deprivation, characterized by an increased AMP/ATP ratio, activates AMPK, which subsequently phosphorylates the activation site Ser555 of ULK1 kinase, hence facilitating autophagosome nucleation and particularly activating mitophagy [55]. Moreover, AMPK alleviates the inhibition of ULK1 by mTORC1 via the phosphorylation of Raptor (a subunit of mTORC1), so creating a positive feedback amplification loop along the AMPK-ULK1 pathway [56]. A recent study indicates that AMPK has a unique role in regulating several mitophagy activities. The primary manifestations are as follows. In the non-ubiquitination-dependent mitophagy pathway, AMPK recruits 14-3-3 proteins by phosphorylating the Ser556 and Ser694 residues of ULK1 to inhibit NiX-dependent mitophagy; conversely, in the ubiquitin-dependent mitophagy pathway, AMPK directly phosphorylates Parkin’s Ser108, thereby augmenting Parkin-dependent impaired mitophagy [57]. Additionally, acetyl-CoA is involved in regulating mitophagy. With enough nutrition, the LRR domain of the endogenous ligand of acetyl-CoA, the mitochondrial NOD-like receptor NLRX1, binds to acetyl-CoA, maintaining it in a self-inhibitory closed conformation and obscuring the LIR motif to obstruct mitophagy. During hunger, NLRX1 oligomerizes, hence diminishing its affinity for acetyl-coenzyme. This leads to LIR exposure, which subsequently attracts LC3 and initiates autophagy [57].

### 3.5. Mitocytosis

Mitocytosis, a distinctive method of mitochondrial secretion, was first identified by Yu Li’s team at Tsinghua University in 2021. It refers to the process by which migratory cells selectively eliminate defective mitochondria through migrasome formation. Mitocytosis primarily entails the selective transport of dysfunctional mitochondria and the necessary positioning of the cytoskeleton. The principal procedure is as follows. Damaged mitochondria are conveyed to the cell periphery by the microtubule motor protein KIF5B and are associated with the cortical actin network via myosin 19, situating them adjacent to the site of migration body formation. The mitochondrial tubular structures extending to the cell membrane are cleaved by DRP1, producing migrasomes that encapsulate fragmented mitochondria around 240 nanometers in diameter, which are then expelled from the cell and disseminated throughout the body [58,59]. This process contrasts with the intracellular autophagosome-lysosome degradation pathway reliant on mitochondrial autophagy; rather, it alleviates the accumulation of damaged mitochondria within cells via a coordinated mechanism of “migration structure-extracellular release”, thus preserving mitochondrial membrane potential, respiratory function, and cellular viability. It is especially appropriate for cell types exhibiting significant migratory activity, such as neutrophils [60,61].

The mitocytosis selection mechanism depends on the recognition and translocation of impaired mitochondria. Mitochondria with decreased membrane potential and increased ROS levels are more easily translocated to the cell periphery and integrated into migrasomes [58]. In this mechanism, DRP1 segregates injured mitochondria from the mitochondrial network; thereafter, kinesin family member 5B (KIF5B) conveys them to the cell membrane, where myosin 19 (Myo19) aids in their localization to cortex-rich areas. Consequently, the execution of mitocytosis necessitates synchronized interactions among mitochondrial dynamics, motor protein transport, and migrasome biogenesis [62].

The interaction between integrins and particular extracellular matrix dictates the localization and efficacy of migratory bodies. The interaction between integrin α5 and fibronectin is the most thoroughly defined model to date [63,64]. The transmembrane protein TSPAN4 and cholesterol are essential for the excretion of migratory bodies: TSPAN4 enhances the aggregation of membrane domains and preserves membrane structural integrity, while cholesterol aids in cell membrane fusion; collectively, they facilitate the formation of retracted fibers [61,65,66].

Recent studies have elucidated that the initiation of migratory bodies is meticulously regulated by lipid and calcium signaling: sphingolipid synthase 2 (SMS2) establishes a fixed focal point at the leading edge of migrating cells, facilitating migratory body growth by converting ceramide to sphingolipids; phosphatidylinositol-4-phosphate 5-kinase 1α (PIP5K1A) synthesizes phosphatidylinositol-4,5-diphosphate (PI(4,5)P_2_) at the formation site, which recruits Rab35, thereby creating a platform for subsequent tetraspanin-dependent expansion through interaction with integrin α5 [67]. The calcium ion (Ca^2+^) sensor synaptotagmin-1 localizes to the migratory body formation site prior to TSPAN4 recruitment, inducing unstable membrane bulging, which is subsequently stabilized in sequence by tetraspanins [68].

## 4. The Deterioration of Mitochondrial Quality Control: A Primary Catalyst of Aging

**Figure 2:**
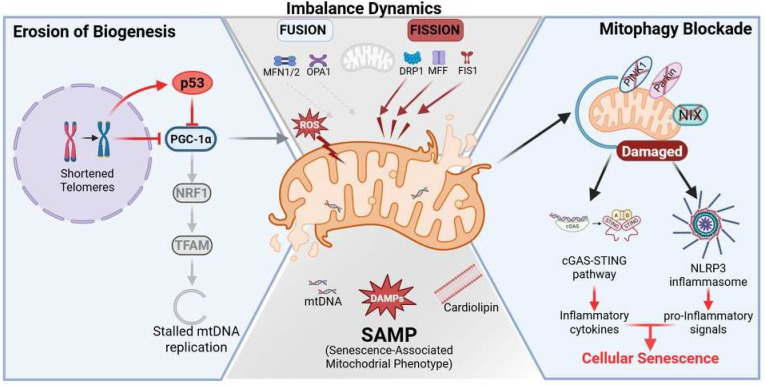
The disruption of the mitochondrial quality control network is a crucial biological mechanism that drives cellular senescence. With aging, mitochondrial homeostasis progressively deteriorates, intensifying the advancement of cellular senescence. Telomere erosion activates the p53 protein, which subsequently inhibits the expression or activity of PGC-1α. Following the functional decline of PGC-1α, its downstream targets NRF1 and TFAM exhibit diminished activity, thereby suppressing mtDNA replication and impairing mitochondrial biogenesis. Furthermore, aging disturbs the dynamic balance between mitochondrial fusion (MFN1/2, OPA1) and fission (DRP1, MFF, FIS1), resulting in the overproduction of ROS and facilitating the release of damage-associated molecular patterns (DAMPs), including free mtDNA and cardiolipin, into the cytoplasm, thereby hastening cellular senescence. Ultimately, compromised mitochondrial autophagy with aging leads to the abnormal accumulation of dysfunctional mitochondria; these inadequately removed damaged mitochondria subsequently initiate an inflammatory cascade that propels the advancement of cellular senescence. Created in BioRender (2026). 18 June 2026. https://BioRender.com/5t6ya9a.

Aging signifies a progressive, systemic decline in physiological integrity, marked by a decrease in the organism’s intrinsic regenerative capacity and an increased vulnerability to degenerative diseases [69]. The systemic breakdown of the MQC network serves as a fundamental molecular mechanism influencing the processes of physiological and pathological aging [70,71]. The disruption of MQC triggers a multi-tiered cascade of failures, resulting in the accumulation of damaged mitochondria and the onset of cellular senescence (Figure 2).

### 4.1. The Deterioration of Biogenesis: A Systemic Failure in Nuclear-Mitochondrial Communication

The decline in mitochondrial biogenesis with aging is not merely due to a decrease in mitochondrial numbers, but rather results from the dysregulation of the mitochondrial renewal system, which includes impaired mitochondrial biogenesis, ineffective clearance of damaged mitochondria, and asynchrony in bidirectional nuclear-mitochondrial communication [72,73]. Under typical circumstances, cells regulate mitochondrial biogenesis by integrating nutritional status, energy stress, redox state, and mitochondrial gene expression via the AMPK–SIRT1/SIRT3–PGC-1α–NRF1/2–TFAM pathway. Nevertheless, certain variables associated with aging can interfere with this process. Research has shown that elevated production of NAD^+^-depleting enzymes such CD38 results in reduced NAD^+^ levels, thereby impairing SIRT1/SIRT3 action [74]. Moreover, DNA damage and telomere attrition associated with aging activate p53, thereby inhibiting PGC-1α production [75,76]. Decreased PGC-1α levels, as a fundamental element of mitochondrial biogenesis, expedite vascular aging and neural metabolic impairment [72,73,77]. Ultimately, chronic inflammation, dysregulated nutrition sensing, and sedentary behavior in aging hinder AMPK’s responsiveness to energy cues, hence further suppressing PGC-1α production. Caloric restriction successfully alleviates hepatic aging, whereas exercise partially reinstates the reduction in PGC-1α-mediated mitochondrial translation and rescues reduced respiratory chain activity in old skeletal muscles [78,79,80]. Consequently, diminished mitochondrial biogenesis with aging contributes to the aging process via many methods. The reduction in mitochondrial quantity results in insufficient ATP synthesis and inadequate respiratory reserves in cells, significantly compromising high-energy-demanding tissues such as skeletal muscle, heart, neurons, and renal tubules [77,81]. Conversely, compromised mitochondrial biogenesis can hinder the respiratory chain, resulting in electron leakage and the excessive production of reactive oxygen species, thereby further undermining the mitochondrial biogenesis process [82]. Mitochondrial biogenesis depends on autophagy for the prompt removal of damaged mitochondria. With aging, reduced autophagy and inadequate mitochondrial synthesis lead to the buildup of impaired mitochondria. Damaged mitochondria produce mtDNA and cardiolipin, subsequently activating pathways such cGAS-STING and inflammasomes, hence intensifying chronic inflammation in senescent cells [83,84].

### 4.2. Stagnant Dynamics: The Senescence-Associated Mitochondrial Dysfunction

The senescence-associated systemic dysregulation of mitochondrial dynamics, leads to the fragmentation of mitochondrial networks and the deterioration of cristae structure. This structural degradation ultimately leads to respiratory chain failure, hence intensifying cellular senescence and overall organismal decline [85,86,87].

Studies indicate that in senescent tissues caused by oxidative stress, calcium overload, inflammatory neurodegenerative disorders, and metabolic diseases, the expression levels of the outer mitochondrial membrane proteins MFN1 and MFN2, as well as the inner membrane protein OPA1, are significantly reduced, while the activity of DRP1 is significantly enhanced, leading to the transformation of mitochondria in cells from tubular structures into short, round fragmented forms. This change results in reduced efficiency of the mitochondrial respiratory chain, increased ROS levels, disruption of calcium homeostasis, and heightened sensitivity to cellular inflammatory responses [85,86,88]. Studies have demonstrated that decreased OPA1 expression levels lead to mitochondrial dynamics imbalance, exacerbating age-related hearing loss [89]. Moreover, OPA1 deficiency is associated with muscle loss; muscle-specific knockout of OPA1 in adult mice induces muscle atrophy, metabolic dysregulation, systemic inflammation, epithelial cell senescence, and a prolonged lifespan phenotype [90]. However, mitochondria in certain aged cells or tissues exhibit excessive fusion, impairing their efficient clearance and subsequently elevating ROS levels while promoting aging-related phenotypes [91]. Research further reveals that DRP1 deficiency in mouse hearts and brains results in increased mitochondrial connectivity, impaired mitochondrial respiration, heart failure, and neurodegenerative damage [92]. However, age-related disparities in mitochondrial fusion and fission do not occur in isolation. They are involved in other mitochondrial functions as well as create a highly synchronized, deleterious cycle characterized by impaired mitophagy, diminished biogenesis, and disturbed proteostasis. Research indicates that the co-deficiency of DRP1 and Parkin in the mouse heart and brain further reduces mitochondrial degradation, thereby exacerbating heart failure and neurodegenerative damage [92], and neurons derived from older donors have diminished membrane potential and altered mitochondrial dynamics. This arises mostly from impaired autolysosomal acidification and reduced dendritic lysosomal reserves, which hinder the removal of defective mitochondria. Pharmacologically enhancing autophagosome production substantially mitigates these hyperactive dynamics and improves mitochondrial health [93].

Disrupted mitochondrial dynamics influence numerous cellular activities associated with the aging process. Mitochondrial dynamic imbalance enhances electron transport chain leakage, raises ROS levels, and disrupts protein homeostasis. Research in Drosophila has shown that increasing DRP1 expression in middle-aged subjects enhances mitochondrial respiration and diminishes protein aggregation in old musculature [94]. Mitochondrial dynamic imbalance consistently triggers inflammatory pathways via the release of mtDNA and cardiolipin while simultaneously inhibiting mitochondrial autophagy, hence exacerbating cellular inflammatory responses [91]. Consequently, mitochondrial dynamic imbalance signifies not only a chemical modification but also an irreversible failure of the entire regulatory network.

### 4.3. Inhibition of Mitophagy: The Accumulation of Proteotoxic Waste and Inflammaging

Mitophagy demonstrates a significant functional decline throughout the aging period. This reduction signifies a dual pathology involving the ubiquitin-dependent system (PINK1-Parkin axis) and the ubiquitin-independent route (receptor-mediated pathway), governed by multi-tiered regulation that includes transcriptional regulation, metabolic signaling, and organelle interaction networks [95,96].

The effectiveness of mitophagy significantly declines with age. Investigations into the ubiquitin-dependent system (PINK1-Parkin axis) have revealed that the absence of PINK1 and Parkin in Drosophila intestinal stem cells leads to a marked increase in age-related markers (elevated ROS levels). The overexpression of PINK1 or Parkin can significantly reduce molecular and biochemical markers of aging, hence prolonging the lifespan of fruit flies. Moreover, in mammals, the expression levels of mRNA and protein for PINK1 and Parkin in the auditory cortex of aged mice were significantly diminished, and the expression level of Parkin in the atrophied muscles of elderly adults was significantly reduced [97,98,99,100]. The reduction in expression of the non-ubiquitin-dependent mitophagy receptor BNIP3L/NIX during human epidermal aging hinders mitophagy in terminally differentiated keratinocytes, leading to the accumulation of damaged mitochondria and diminished ATP-dependent mitochondrial respiratory function, which exacerbates and aggravates cellular senescence [101]. Impaired mitophagy with aging leads to the accumulation of dysfunctional mitochondria, ensnaring cells in a self-sustaining loop of mitochondrial stress, ROS bursts, and progressive senescence. The continuous emission of ROS from these depolarized organelles accelerates oxidative damage and mitochondrial DNA mutagenesis while also inducing the cytosolic release of mitochondrial DNA. This directly activates the cGAS-STING and NLRP3 inflammasome pathways, hence augmenting the senescence phenotype of cells [96]. This defective clearance process is consistently linked to systemic disorders, such as Aβ- and p-tau-induced neurotoxicity in Alzheimer’s disease, along with insulin resistance and lipid dysregulation in metabolic syndromes [96,102].

## 5. MQC Dysregulation: A Contributor to Age-Related Pathology

The dysfunction of the MQC regulatory network is a crucial factor contributing to the pathophysiology of age-related illnesses. The integrity of this network relies on the meticulous regulation of mitochondrial synthesis, homeostasis, and autophagy. Systemic dysregulation leads to the accumulation of dysfunctional mitochondria, exacerbation of oxidative stress, and disruptions in energy metabolism, which have been identified as a common pathological basis for neurodegenerative diseases, cardiovascular diseases, metabolic disorders, and cancer [103,104]. This section will elucidate the pathogenic pathways of mitochondrial dysfunction across various physiological states. Firstly, the mitochondrial dysfunction in senescent tissues leads to a bioenergetic deficit in high-energy metabolic tissues; secondly, there is systemic metabolic rigidity affecting glucose and skeletal homeostasis; additionally, malignant tumors exhibit a paradox wherein cancer cells aberrantly utilize the mitochondrial quality control mechanism in response to a deteriorating host environment, thereby promoting their unchecked proliferation. However, the majority of evidence connecting MQC dysfunction to this disease originates from cellular or animal models, and disease-specific biomarkers indicative of MQC activity in humans are inadequately verified.

### 5.1. The Bioenergetic Crisis: Neurodegeneration and Cardiovascular Dysfunction

The progressive deterioration of the MQC system serves as the primary biological mechanism linking cardiovascular and cerebrovascular aging to disease progression. Neurons and cardiomyocytes, as energy-demanding cells, fundamentally rely on the integrity of a multi-tiered mitochondrial quality control network for their survival and functional maintenance [85,105,106].

#### 5.1.1. Neurodegenerative Diseases

PGC-1α, a major regulatory factor in mitochondrial biogenesis, is highly expressed in the central nervous system [107]. In neurodegenerative diseases associated with aging, the expression level of PGC-1α is markedly diminished, which subsequently impedes the transcription of downstream NRF1/2-TFAM and the production of oxidative phosphorylation subunits, thereby instigating a cascade of events such as mtDNA depletion, contributing to disease advancement [108]. The phenomena have been validated in several neurological disorders, including Alzheimer’s disease, Parkinson’s disease, Huntington’s disease, and amyotrophic lateral sclerosis [107].

In contrast to the mitochondrial fusion rate mediated by MFN1/2-OPA1, senescent neurons demonstrated a markedly increased rate of mitochondrial fission induced by DRP1. This leads to mitochondrial network fragmentation, structural impairment to the cristae, and dissociation of the respiratory chain [94,108]. Neuronal synapses, being specialized organelles, rely on mitochondria for energy supply and calcium regulation during synaptic transmission. Research indicates that the extent of mitochondrial damage in synapses is strongly inversely connected with aging, a conclusion corroborated in persons with Alzheimer’s disease, Parkinson’s disease, and Huntington’s disease [109]. Subsequent study indicates that senescent astrocytes exhibit mitochondria fragmentation, a reduction in mitochondrial membrane potential, and a decrease in the expression of genes related to mitochondrial biosynthesis (PGC-1α, TFB1, ATP5A1) [108].

Investigations into models of aging-associated neurodegenerative disorders have demonstrated that PINK1-Parkin-mediated ubiquitin-dependent mitophagy is suppressed. In Parkinson’s disease, dysfunctions in the PINK1/PARKIN signaling cascade result in insufficient tagging and the elimination of impaired mitochondria. The accumulation of depolarized mitochondria promotes the persistent production of ROS, ultimately leading to cellular dysfunction [110,111]. Studies on Alzheimer’s disease models reveal that the downregulation of mitophagy receptors (OPTN, NDP52) and lysosomal dysfunction lead to impaired autophagy flux, which intensifies mitochondrial damage caused by Aβ and phosphorylated tau, thereby hastening disease progression [110]. Recent studies indicate that NAD+ enhancers, such as NMN, augment activated mitophagy and unfolded protein response pathways in models of Parkinson’s disease [112]. These findings indicate that mitochondrial dysfunction is a significant causal element in the advancement of neurodegenerative disorders. Alleviating this functional anomaly may hinder the progression of various illnesses.

#### 5.1.2. Cardiovascular Disorders

Dysregulation of mitochondrial quality control mechanisms plays a significant role in the pathophysiology of cardiovascular diseases, primarily marked by the release of excessive mtDNA and cardiolipin from impaired mitochondria. The released mtDNA and cardiolipin activate the cGAS-STING and NLRP3 inflammasomes, leading to aseptic inflammation within cells [113,114]. In the interim, mitochondrial dysfunction results in inadequate intracellular ATP production, which ultimately impairs cardiac contractile function [113]. Moreover, the clinical symptoms of cardiac disorders may be intensified by mitochondrial dysfunction, which can directly provoke myocardial cell hypertrophy, myocardial fibrosis, and ventricular remodeling [105].

The expression of PGC-1α, an essential protein in mitochondrial biogenesis, diminishes with aging in heart tissue. This results in a decrease in the amount of mitochondrial DNA (mtDNA) and impairs the production of oxidative phosphorylation proteins. Investigations utilizing PGC-1α-deficient animals to assess the impact of mitochondrial biogenesis on cardiac function revealed that these mice exhibited considerable myocardial dysfunction under conditions of stress overload [113]. Furthermore, the mitochondrial homeostatic imbalance associated with SIRT1/PGC-1α/Mfn2 plays a role in age-related vascular calcification [115]. Research demonstrates that in the cardiac tissue of 24-month-old mice, the expression levels of mitochondrial fusion-related genes (MFN1, MFN2, and OPA1) are significantly reduced, whereas the expression levels of mitochondrial fission-related genes (FIS1 and DRP1) are considerably increased [116]. In studies of a high-fat diet-induced obese cardiomyopathy model, researchers observed a significant increase of DRP1 (a mitochondrial fission protein) expression in cardiomyocytes, accompanied by a marked downregulation of MFN1/2 (proteins associated with mitochondrial fusion). These modifications result in impaired heart metabolic function [116]. Furthermore, studies have demonstrated that reduction or deficiency in OPA1 disrupts mitochondrial structure and impairs the respiratory chain function, thereby decreasing ATP production in cardiomyocytes and increasing the risk of cytochrome C release [117]. The aforementioned suggests that mitochondrial dynamic equilibrium influences cardiac cell senescence, with obesity exacerbating this effect. In aged cardiomyocytes, the expression levels of mitophagy receptors (OPTN, NDP52) are significantly reduced, leading to the accumulation of depolarized mitochondria, which further exacerbates cardiomyocyte senescence [118], while enhancing Parkin-mediated mitochondrial autophagy in the heart can improve mitochondrial health and delay cardiac aging [105]. The data indicate that mitochondrial dysfunction is a key component contributing to the progression of cardiac problems in aging persons. Maintaining MQC homeostasis may potentially delay the start and progression of cardiac diseases with aging.

### 5.2. Metabolic Disorders: Systemic Insulin Resistance and Skeletal Fragility

The dysregulation of MQC in essential metabolic organs, such as pancreatic β-cells, liver, skeletal muscle, and adipose tissue, can lead to impairments in insulin production and insulin resistance as a result of aging. This cascade leads to energy metabolism abnormalities, increased oxidative stress, and cellular senescence, ultimately promoting the progression of type 2 diabetes and skeletal system illnesses [119].

#### 5.2.1. Type 2 Diabetes

The expression of PGC-1α, an essential protein in mitochondrial biogenesis, declines with age, leading to compromised oxidative phosphorylation and contributing to abnormal glucose metabolic responses in the body. Clinical research involving patients with type 2 diabetes mellitus (T2DM) and prediabetic individuals has demonstrated that PGC-1α expression is simultaneously reduced in skeletal muscle cells and pancreatic β-cells, resulting in disturbances in cellular energy metabolism and insufficient insulin production [120]. Furthermore, research on pancreatic β-cells, hepatocytes, and brown adipocytes has revealed that deficiencies in MQC trigger mitochondrial integrated stress responses through the activation of the mitochondrial-nuclear signaling pathway, leading to chromatin remodeling and the degradation of cellular identity characteristics [121].

The dynamic equilibrium of mitochondria is essential for the insulin release in pancreatic β-cells. Research reveals that the limitation of mitochondrial fission reduces glucose sensitivity in β-cells, leading to impaired glucose regulation. Investigations concerning high-glucose treatment of β-cells revealed a marked reduction in the expression of the mitochondrial fusion protein MFN2, while the expression of the mitochondrial fission protein DRP1 was considerably increased. This led to excessive mitochondrial fission, resulting in inadequate ATP generation in cells, which impaired insulin release by β-cells and altered glucose homeostasis in the body. Furthermore, research on β-cell-specific Drp1 knockout mice demonstrated increased mitochondrial fusion in β-cells, accompanied by disturbance in calcium signaling and impaired insulin synthesis. The results demonstrate that mitochondrial fission is essential for the physiological response to glucose [122]. Moreover, the PINK1-Parkin pathway-mediated ubiquitin-dependent mitophagy flux is significantly reduced in age-related T2DM, leading to the accumulation of depolarized mitochondria and continuous production of ROS. Elevated ROS induce concurrent oxidative stress and inflammation in pancreatic β-cells, ultimately resulting in diminished insulin synthesis [123]. Furthermore, dysfunctional mitochondria release increased quantities of mtDNA and cardiolipin into the cytoplasm. Mitochondrial DNA and cardiolipin within the cell activate the NLRP3 inflammasome, leading to aseptic inflammation and ultimately creating a harmful cycle of ‘mitochondrial–inflammation–diabetes’ [124]. Mitochondrial dysfunction is a primary factor in the failure of pancreatic β-cells throughout aging. Restoring mitochondrial homeostasis may mitigate the decline of pancreatic β-cell facticity associated with aging.

#### 5.2.2. Skeletal System Diseases

The deterioration of the skeletal system mostly manifests as sarcopenia, osteoporosis, and osteoarthritis, which are the principal factors leading to functional impairment and diminished quality of life in the elderly population [125].

Research on sarcopenia reveals that affected individuals exhibit significantly reduced expression of PGC-1α in skeletal muscle cells, leading to compromised mitochondrial biosynthesis, which exacerbates muscle energy imbalance and accelerates the onset of muscle fiber atrophy symptoms [126]. Moreover, research on aged skeletal muscle cells indicates less mitochondrial fusion and increased mitochondrial fission. Simultaneously, the PINK1-Parkin-mediated ubiquitin-dependent mitophagy pathway is concurrently impaired, resulting in insufficient elimination of damaged mitochondria, which accelerates the senescence of skeletal muscle cells [126].

The mitochondrial quality of bone-associated cells is essential for maintaining the balance of bone remodeling in osteoporosis [127]. The osteogenic differentiation of bone marrow mesenchymal stem cells (BMMSCs) is profoundly affected by mitochondrial activity and mtDNA amount, as demonstrated by clinical research. The osteogenic differentiation potential of MSCs is compromised by diminished mitochondrial activity and mtDNA levels. Osteoclast-specific TFAM gene knockout mice were used in a study to illustrate that osteoclast activity escalates with age, leading to substantial bone loss in these mice (NCT05483738). The aforementioned findings indicate significant potential for addressing mitochondrial quality control to mitigate age-related osteoporosis. Recent research has confirmed that genistein can alleviate senescence characteristics in BMMSCs of ovariectomized mice by modulating mitochondrial biosynthesis and mitophagy activities [128].

Impairment of MQC in chondrocytes leads to chondrocyte senescence and worsens osteoarthritis symptoms. The expression level of MFN2 in chondrocytes is markedly diminished in osteoarthritis, as demonstrated by study findings [129]. The advancement of osteoarthritis can be mitigated by reinstating MFN2 expression levels, while the depletion of MFN2 hastens chondrocyte senescence via impairing calcium homeostasis in chondrocytes [130]. Mitophagy is crucial in osteoarthritis. The lack of PINK1 exacerbates degenerative changes in cartilage tissue, while PINK1 overexpression alleviates chondrocyte senescence via inhibiting the p38 MAPK/NF-κB signaling pathway [131]. The observations indicate that maintaining normal mitochondrial function in chondrocytes during aging may significantly mitigate the incidence of osteoarthritis. A recent study demonstrates that in an IL-1β-induced osteoarthritis chondrocyte model, ginsenoside Rh1 can restore mitophagy in chondrocytes via activating the AMPK/PINK1/Parkin pathway, hence reducing chondrocyte senescence and extracellular matrix degradation [132].

### 5.3. The Oncogenic Paradox: Maladaptive Exploitation of MQC for Survival

The disturbance of the MQC regulatory network plays a dual role in cancer. Unlike degenerative illnesses caused by MQC failure, tumor cells utilize the MQC route through adaptive reprogramming, transforming it into a mechanism for acquiring survival advantages and therapeutic resistance [103,133].

PGC-1α expression is significantly reduced in senescent tissues, whereas it exhibits an inverse trend in tumor cells. Studies demonstrate that increased PGC-1α levels in circulating tumor cells (CTCs) enhance tumor cell invasion and distant metastasis via promoting oxidative phosphorylation and mitochondrial biogenesis. Suppression of PGC-1α expression in cancer cells can reduce their invasive and metastatic potential [134]. Similarly, as age advances, the suppression of mitophagy in cancer cells has a dual role in tumor growth. In the initial phases of tumor development, inadequacies in mitophagy lead to the accumulation of impaired mitochondria and sustained generation of ROS, significantly increasing the likelihood of normal cells transforming into cancerous cells [135]. Furthermore, specific autophagy-related proteins have tumor-suppressive characteristics; for instance, the lack of BNIP3 enhances development and metastasis in breast cancer [136]. Conversely, several chemotherapy agents (e.g., doxorubicin and cisplatin) induce mitochondrial dysfunction and oxidative stress to eradicate cancer cells. Studies indicate that enhancing mitophagy by suppressing the BNIP3 gene in rectal cancer stem cells elevates their vulnerability to doxorubicin [137]. In transformed cells, the disruption of mitophagy may impede tumor growth by inducing cell cycle arrest and apoptosis [138,139]. However, KRAS inhibitor therapy can stimulate the NLRX1-mediated mitophagy pathway, enabling tumor cells to evade drug-induced cytotoxicity and acquire chemoresistance [140]. This underscores the complexity of mitophagy regulation within the tumor microenvironment.

## 6. Therapeutic Frontiers: Targeting MQC for Geroprotection and Disease Reversal

Recent research on aging has shifted from regarding mitochondrial failure as the exclusive marker of aging to recognizing MQC dysfunction as the primary driver of organismal aging. Consequently, methods for delaying aging have evolved from rudimentary antioxidant supplements to more targeted therapies. Concentrating on the regulatory mechanisms that oversee mitochondrial quality holds significant potential for anti-aging interventions. This section examines several methods to modify mitochondrial activity to decelerate aging, including lifestyle alterations and novel pharmaceuticals.

### 6.1. Lifestyle Interventions

Physical activity and dietary adjustments are universally recognized as crucial for prolonging a healthy lifespan; their effectiveness is attributed not only to metabolic enhancements, but also to the activation of the evolutionarily conserved MQC signaling pathway. A five-year longitudinal study including 500,000 individuals showed that adverse living conditions, lifestyles, and social pressures reduce mitochondrial quantity to varying degrees and increase mitochondrial susceptibility to aging [141]. Two further investigations including the elderly revealed that regardless of genetic predispositions linked to aging, a nutritious diet and regular physical activity could alleviate hereditary risks and significantly improve healthy life expectancy [142]. In excitatory tissues such as heart, skeletal muscle, and the central nervous system (CNS), these therapies enhance mitochondrial plasticity through stress signals, hence increasing resilience against protein toxicity and oxidative stress.

#### 6.1.1. Exercise

Exercise, as a non-pharmacological method to restore MQC homeostasis in aging tissues, has demonstrated significant therapeutic efficacy. Research indicates that persons who engage in regular physical activity as they age maintain mitochondrial respiratory capacity in skeletal muscles, whereas sedentary individuals have a marked decline in this function. This discovery suggests that exercise can delay muscle aging by maintaining the muscular biosynthesis process [143,144], and a meta-analysis on exercise and skeletal muscle mitochondrial biogenesis found PGC-1α as the most frequently reported biomarker of mitochondrial biogenesis in exercise regimens [145]. Moreover, a 16-week aerobic exercise program in typically aged mice resulted in significant increases in the expression of genes and proteins associated with mitochondrial quality control, enhancing skeletal muscle strength through increased mitochondrial respiratory capacity [146]. Results from human skeletal muscle biopsies indicate that irrespective of age (young or elderly), persons participating in prolonged exercise display increased FIS1 expression in skeletal muscles, as well as heightened levels of fusion and oxidative phosphorylation proteins. Furthermore, aged adults who engage in exercise exhibit elevated levels of Parkin expression relative to their sedentary counterparts, suggesting that consistent physical activity aids in the preservation of mitochondrial quality control throughout life [147]. Subsequent clinical investigations have demonstrated that high-intensity regular exercise can mitigate age-associated reductions in mitochondrial capacity, mitochondrial protein synthesis, exercise performance, muscle function, and insulin sensitivity [81,148,149]. These advantages are chiefly ascribed to exercise’s modulation of systemic inflammatory conditions through circulating molecules (e.g., peptides, metabolites, and nucleic acids), consequently improving mitochondrial homeostasis. Clinical data studies indicate that long-term exercisers exhibit a biological age approximately 5.8 years younger than their peers [150,151,152].

#### 6.1.2. Dietary Adjustments

Excessive feeding can expose cells to sustained conditions of elevated energy, sugar, and fat levels. This promotes the continuous stimulation of protein synthesis, lipid synthesis, and cell proliferation, while simultaneously inhibiting autophagy and damage clearance pathways, ultimately resulting in the failure of cellular nutrition sensing mechanisms. This pathogenic mechanism is crucial in regulating the development of age-related illnesses. The swift advancement of age-related illnesses is frequently due not to inadequate energy availability, but to functional impairments in energy consumption processes. The enduring nature of this clinical condition exacerbates chronic inflammatory responses, hence hastening the aging process. Furthermore, prolonged nutritional surplus leads to adipocyte hypertrophy, facilitates hypoxia, triggers cell death, and results in immune cell infiltration inside adipose tissue, while also steering macrophages toward a pro-inflammatory phenotype. Simultaneously, the body compensates by secreting extra insulin to preserve glucose homeostasis; the combined impact of heightened insulin levels and chronic inflammation throughout various organs can lead to insulin resistance. Chronic overstimulation adversely affects pancreatic β-cell function, hence hastening the onset and progression of type 2 diabetes. Moreover, under states of nutritional surplus, mitochondria experience elevated levels of fatty acids and glucose substrates, resulting in heightened generation of ROS. Failure of mitochondrial quality control initiates a cascade of inflammatory responses that expedite cellular aging and ultimately lead to severe chronic metabolic diseases. In conclusion, the significance of calorie restriction in postponing the advancement of age-related disorders is becoming increasingly apparent [70,153,154,155].

Caloric restriction (CR) involves a reduction in caloric intake by 20–40% without causing malnutrition and is currently considered one of the most effective interventions for delaying aging [156]. The conventional perspective posits that caloric restriction stimulates mitochondrial biogenesis through the activation of the SIRT1–PGC-1α–NRF1/2–TFAM pathway, which increases TFAM expression, augments mitochondrial protein synthesis and oxidative phosphorylation, and ultimately enhances mitochondrial renewal [157,158]. Preliminary human research has corroborated this conventional perspective. Following a six-month intervention involving 36 young, overweight yet non-obese adults, findings revealed enhanced expression of mitochondrial function-associated genes, including PGC-1α, TFAM, eNOS, SIRT1, and PARL in skeletal muscle among the CR and CR+ exercise cohorts; mitochondrial DNA (mtDNA) content increased by approximately 35% in the CR group and by about 21% in the CR+ exercise group; concurrently, muscle DNA damage was diminished [159]. Conversely, alternative research contends that caloric restriction does not directly stimulate mitochondrial synthesis; instead, it augments mitochondrial function by preserving existing mitochondrial elements, enhancing antioxidant clearance capabilities, and diminishing oxidative damage levels. This conclusion was subsequently corroborated in mice subjected to lifelong caloric restriction, where researchers discovered that such restriction preserved the oxidative capacity and efficiency of skeletal muscle mitochondria in elderly mice without augmenting mitochondrial quantity [160]. Secondly, caloric restriction can reestablish the dynamic equilibrium of mitochondria and the association with autophagy. Research has shown that caloric restriction diminishes increased concentrations of MFN2 and FIS1 in the gastrocnemius muscle of aged mice [160]. Recent studies demonstrate that mild fasting and other shortages decrease cytoplasmic acetyl-CoA levels, hence reducing its affinity for the LRR domain of mitochondrial NLRX1. This method facilitates the recruitment of LC3 protein and initiates the selective elimination of damaged mitochondria, hence diminishing free radical generation to maintain cellular viability [156]. Nevertheless, the majority of existing scientific evidence regarding heat restriction-induced mitochondrial autophagy is derived from animal and cellular studies, with scant data accessible from higher animals and clinical investigations [161]. Moreover, calorie restriction can diminish DNA damage in human muscle tissue [159] and reduce oxidative stress in mice, thereby alleviating both DNA damage and protein oxidative damage [162]. Recent human investigations indicate that a caloric restriction of around 14% sustained over two years can improve thymusogenesis [163], and that caloric restriction diminishes several indicators linked to cellular aging [164]. Consequently, caloric restriction preserves mitochondrial quality control not via a singular channel, but by enhancing mitochondrial function through various mechanisms. Moreover, alongside calorie restriction, dietary alterations can enhance mitochondrial quality control. Phenolic compounds enhance mitochondrial activity by neutralizing free radicals and reinstating the antioxidant enzyme system. Epidemiological evidence indicates that consistent adherence to a phenol-rich dietary pattern—characterized by a high consumption of fruits, vegetables, nuts, legumes, and unsaturated fatty acids—significantly correlates with healthy aging after 70, defined by the absence of 11 major chronic diseases and the maintenance of cognitive, physical, and mental functions [165].

### 6.2. Pharmacological Geroprotectors

A healthy lifestyle supports anti-aging; nonetheless, age-related anabolic resistance needs targeted medicinal interventions to rectify perceived physiological inadequacies. Next-generation therapeutic agents restore normal tissue function by reactivating mitochondrial quality control mechanisms that gradually become inactive with aging.

#### 6.2.1. Pharmacological Manipulation of Mitochondrial Biogenesis to Mitigate Aging

Pharmaceuticals impede aging by augmenting PGC-1α expression to rejuvenate mitochondrial biosynthesis. Studies indicate that mesoporous silica nanoparticles (SP@NNPm) coated with a PGC-1α inducer (SP) and overexpressed natural killer cell 2 group member D (NKG2D) can effectively stimulate PGC-1α-mediated mitochondrial biosynthesis in aging models, thereby alleviating cellular senescence characteristics and postponing the degenerative progression of intervertebral discs [166]. Furthermore, metformin promotes mitochondrial biogenesis via the activation of the AMPK-PGC-1α pathway, hence augmenting energy metabolism. Consequently, metformin is seen as a promising medication with possible anti-aging effects [167].

#### 6.2.2. Pharmacological Modulation of Mitochondrial Dynamics to Mitigate Aging

Mitochondria in senescent cells generally display excessive fission or irregular fusion, leading to collapse of the mitochondrial network and subsequent mitochondrial dysfunction. Pharmacological intervention targeting mitochondria to restore their dynamic equilibrium is an effective approach to delay tissue aging. The DRP1 inhibitor Mdivi-1 has demonstrated the ability to obstruct high-glucose-induced senescence in vascular endothelial cells by inhibiting the cGAS-STING pathway and subsequent generation of inflammatory cytokines in these cells [168]. Additionally, flavonoids and homocyanidin (Hom) derived from sage inhibit the activation of the cGAS-STING pathway by preventing the translocation of DRP1 into mitochondria and subsequent mitochondrial fission, hence alleviating inflammation-induced cellular senescence [168].

#### 6.2.3. Pharmacological Modulation of Mitophagy to Prolong Longevity

Mitophagy is a crucial quality control mechanism for the elimination of impaired mitochondria, and its functional decline is the principal cause of mitochondrial damage accumulation with aging. Studies indicate that in aging models of high-fat diet-induced cochlear cells (HEI-OC1) and cochlear explants, the SIRT1 agonist SRT2104 safeguards mitochondrial function by upregulating the expression of mitophagy-related proteins (including PINK1, Parkin, BNIP3, and LC3-II), consequently reducing the levels of aging markers (p53, p21) and the number of aging-associated β-galactosidase (SA-β-gal) positive cells. The suppression of SIRT1 completely nullifies these protective effects, indicating that the control of mitophagy may alleviate aging [169]. SIRT3 is a NAD^+^-dependent deacetylase located in the mitochondrial matrix, crucial for maintaining MQC. In the realm of senescence and metabolic strain, terpenoids derived from common herbs (thymol and carvacrol) activate PINK1-dependent mitophagy through their interaction with SIRT3, thereby significantly reducing hepatic lipid accumulation in mice subjected to a high-fat diet, as well as alleviating the functional decline of skeletal muscle and epigenetic aging in SAMP8 mice displaying accelerated senescence [170]. Prior research suggests that pharmaceutical therapies targeting mitochondrial quality control demonstrate significant potential in delayingaging.

### 6.3. Bioengineering and Genetic Technologies for Modulating Mitochondrial Quality Control to Prolong Lifespan

Although pharmacological interventions can affect signaling pathways, they cannot physically replace absent organelles or correct accumulated somatic mutations. To overcome the inherent limitations of small-molecule medicines, organelle transplantation and precise genome editing can markedly improve mitochondrial activity, offering novel therapeutic strategies for aging mitigation.

#### 6.3.1. Mitochondrial Transplantation

The mitochondrial transplantation approach entails the infusion of exogenous healthy mitochondria into cells or tissues exhibiting functional deficiencies, promoting the elimination of defective mitochondria in recipient cells through activation of the PINK1-Parkin-mediated mitophagy pathway. This method significantly enhances the quantity of mitochondria in damaged cells while simultaneously improving their functionality, hence preserving cell viability [171]. Studies indicate that intravenous injection of mitochondria from young mice into older specimens may markedly enhance mitochondrial function, reduce oxidative stress, and perhaps reverse cognitive and physical decline associated with aging [172]. In a 24-month-old aging mouse model, a single allogeneic mitochondrial transplantation into hindlimb muscles (quadriceps, tibialis anterior, and gastrocnemius) resulted in a 65% increase in basal ATP levels, a significant enhancement in cytochrome c oxidase and citrate synthase activities, a twofold increase in mitochondrial marker protein expression levels, and a notable improvement in exercise endurance in recipient mice [173]. The study demonstrated that transferring healthy mitochondria from adipose-derived mesenchymal stem cells (ASCs) of healthy donor horses to recipient ASCs with impaired metabolic efficiency could enhance their ability to alleviate inflammatory responses in synovial cells stimulated by LPA [174]. In a UVA radiation-induced skin photoaging model, mitochondrial transplantation reinstated the oxidative environment of fibroblasts, reduced dermal collagen deployment, increased collagen fiber density, and improved the senescent phenotype of tissue architecture in animal models [175]. The observations suggest that altering the mitochondrial quality control network in impaired cells and tissues through mitochondrial transplantation has considerable application potential for mitigating aging.

#### 6.3.2. Mitochondrial Gene Editing

Mutations in mitochondrial DNA are significantly associated with aging and several degenerative diseases. Globally, around 1 in 5000 persons carries harmful mitochondrial DNA mutations, with 95% of them being point mutations [176]. Nonetheless, traditional nuclear gene editing methods present difficulties when used on mitochondria. Recently proposed DddA-derived cytosine base editors (DdCBEs) utilize the DddA deaminase from bacterial toxins, targeted to the mitochondrial matrix via mitochondrial targeting signals, to enable C•G to T•A conversion without inducing double-strand breaks [177]. Researchers have created mitochondrial adenine base editors (mitoABEs) that enable the conversion of A•T to G•C base pairs. The eTd-mtABE variant series, resulting from the guided evolution of TadA deaminase, has markedly enhanced mitochondrial gene editing efficiency [178,179]. The researchers, led by Li Dali, utilized DdCBE variants in a Leigh disease rat model with near-homoplasmic mutations to improve the recovery rate of wild-type mtDNA to 53%. This significantly improved motor dysfunction and cardiac impairment in the model organisms, restoring functional levels akin to those of wild-type rats [178,179]. Despite considerable progress in mitochondrial genome editing, its therapeutic use faces considerable challenges—specifically, off-target effects (including nuclear genome and mitochondrial bystander editing) require further refinement [177].

Nonetheless, both pharmaceutical therapies and genetic engineering strategies aimed at mitochondrial quality management to mitigate aging have thus far been validated solely at the cellular or animal level. Additional validation in primates and clinical environments is necessary prior to the clinical application of these techniques.

## 7. Conclusions and Future Perspectives

This review consolidates extensive evidence demonstrating that aging may essentially represent a biological energy crisis. Impairments in MQC serve as indicators of aging and as pathogenic catalysts for several age-associated illnesses, including neurological disorders and cardiovascular failure. Nevertheless, several obstacles persist until the successful treatment of aging-related disorders may be realized with focused MQC treatments.

### 7.1. Constraints of Bioavailability

Notwithstanding the significant potential of novel anti-aging drugs in delaying aging by targeting the MQC, their practical application faces tremendous obstacles. The limitations emerge from various systemic issues, including pharmacokinetics, efficacy, safety, and illness complexity.

The principal challenge in the therapeutic application of natural polyphenols is their bioavailability issue. Pharmacokinetic investigations in humans demonstrate that the parent chemical entering systemic circulation after oral treatment exhibits significantly low quantities. The majority of the substances rapidly convert into inactive glucuronides and sulfate conjugates in the liver and intestines. As a result, the dosages employed in in vitro pharmacodynamic investigations are nearly unachievable under physiological settings in humans. Furthermore, the clinically characterized circulating forms are primarily their metabolites, which exhibit significantly different biological action compared to the parent medication [180]. Furthermore, significant interspecific variability poses an additional critical hurdle to the clinical translation of anti-aging therapeutic medicines. NAD+ precursors demonstrated significant anti-aging benefits in preclinical studies; however, human clinical trials failed to replicate the profound phenotypic reversals observed in animal models. These inequities can be attributed to the complexities of metabolic regulatory networks, variations in drug target distribution among tissues, and inconsistencies in necessary threshold values [181,182]. In patients with confirmed monogenic premature aging syndromes, such as Werner syndrome, NAD+ precursor supplements have demonstrated a considerable improvement in arteriosclerosis and skin ulceration [183]. Significantly, contemporary clinical trials predominantly depend on surrogate endpoints (such as insulin sensitivity and inflammatory cytokine levels); the extent to which these metrics can be genuinely correlated with definitive endpoints (e.g., prolonged healthy lifespan and diminished disability rates) remains uncertain [184].

### 7.2. The Imperative of Precision Therapy

At present, no singular independent biomarker can universally detect all senescent cells, suggesting that the nature and extent of mitochondrial dysfunction may vary considerably among persons, tissues, and cells within the same tissue [185]. This heterogeneity is especially evident throughout the aging process [186]. Researchers have uncovered both sex-specific and common molecular fingerprints in frail elderly populations that converge on unique mitochondrial pathways. The development of biomarkers reflecting mitochondrial activity during aging offers a valuable means for the early detection of functional decline and therapeutic approaches.

### 7.3. The Imperative of Liquid Biopsies

Although artificial intelligence models can improve medication screening and precision medicine through predictive analytics, the clinical translation process still relies on invasive biopsies. Invasive biopsies not only cause patient discomfort but also significantly impede the conduct of clinical investigations. Therefore, the development of highly reproducible, cost-effective, and high-throughput diagnostic instruments is crucial for the effective use of a targeted mitochondrial anti-aging treatment. Peripheral blood, serving as a “liquid biopsy sample”, has excellent reproducibility, cost-efficiency, and significant throughput [187]. Contemporary research emphasizes classic indicators like the inducible cytokine GDF15 and the development of innovative detection techniques, including circulating free mitochondrial DNA (cf-mtDNA) and critical mitochondrial proteins found in extracellular vehicles (EVs). Recent research suggest that mitochondrial vesicles may provide a non-invasive method to assess the bioenergetic condition of the brain and heart, facilitating the real-time monitoring of therapy efficacy using simple blood samples [188].

**Figure 3:**
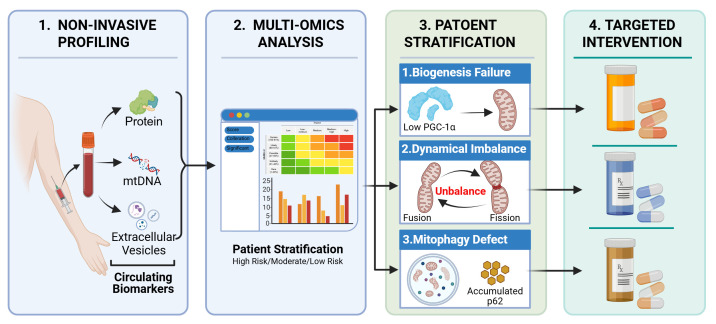
An illustration of the translational research and precision medicine on aging brought on by the dysregulation of MQC. The image portrays a specific therapeutic approach aimed at delaying the aging process. The procedure comprises four fundamental phases. (1) Non-invasive characteristic analysis: Collection of blood samples to measure circulating biomarkers for the evaluation of overall mitochondrial health. (2) Multi-omics analysis: Utilizing high-throughput multi-omics technologies to gather complex data for comprehensive analysis; (3) Classification of individuals into three risk categories (high, medium, or low) based on age-related mitochondrial characteristics; patient stratification employs sophisticated methodologies that integrate molecular pathways regulating mitochondrial quality control with associated risk levels. (4) Targeted intervention strategies utilizing these precise molecular classifications to guide therapeutic application. Created in BioRender (2026). 18 June 2026. https://BioRender.com/8o351it.

Despite the numerous challenges related to the development of the aforementioned technologies, the combined application of these approaches may finally render mitochondrial-targeted anti-aging as a clinically feasible diagnostic and therapeutic tool (Figure 3).

## Data Availability

No new data were created or analyzed in this study. Data sharing is not applicable to this article.

## Abbreviations

The following abbreviations are used in this manuscript:

AMPK	Adenosine 5′-monophosphate (AMP)-activated protein kinase
ARE	Antioxidant response element
ASCs	Adipose-derived mesenchymal stem cells
ATFS-1	Activated transcription factor atfs-1
BMMSCs	Bone marrow mesenchymal stem cells
Ca^2+^	Calcium ions
cf-mtDNA	Circulating free mitochondrial DNA
CLPP	Caseinolytic protease P
CNS	Central nervous system
CR	Caloric restriction
CTCs	Circulating tumor cells
DAMPs	Damage-associated molecular patterns
DdCBEs	DddA-derived cytosine base editors
EVs	Extracellular vehicles
FNIP1	Folliculin interacting protein 1
HEI-OC1	High-fat diet-induced cochlear cells
Hom	Homocyanidin
KIF5B	Kinesin family member 5B
LONP1	Lon peptidase 1
Lys63	Lysine 63
MAMs	Endoplasmic reticulum-mitochondrial contact sites
MFF	Mitochondrial fission factor
MFN1/2	Mitofusin 1/2
mitoABEs	Mitochondrial adenine base editors
MQC	Mitochondrial quality control
MeSH	Medical Subject Headings
mtDNA	Mitochondrial DNA
mTORC1	Mechanistic target of rapamycin complex 1
mtROS	Mitochondrial reactive oxygen species
Myo19	Myosin 19
NAD+	Nicotinamide adenine dinucleotide
NKG2D	Natural killer cell 2 group member D
NRF-1/2	Nuclear respiratory factors 1/2
OPA1	Optic atrophy 1
PGC1α-SIRT1	Peroxisome proliferator-activated receptor γ coactivator 1-alpha-sirtuin 1
PINK1	PTEN-induced kinase 1
PIP5K1A	Phosphatidylinositol-4-phosphate 5-kinase 1α
ROS	Reactive oxygen species
SAMP	Senescence-associated mitochondrial phenotype
SA-β-gal	Aging-associated β-galactosidase
SMS2	Sphingolipid synthase 2
T2DM	Type 2 diabetes mellitus
TFAM	Mitochondrial transcription factor A
TFEB	Transcription factor EB
UPR^mt^	Mitochondrial unfolded protein response
Δψm	Mitochondrial membrane potential
